# Collectanea Medica, Consisting of Anecdotes, Facts, Extracts, Illustrations, Queries, Suggestions, &c.

**Published:** 1817-11

**Authors:** 


### COLLECTANEA MEDICA,

CONSISTING OF

### ANECDOTES, FACTS, EXTRACTS, ILLUSTRATIONS, QUERIES, SUGGESTIONS, &c.

RELATING TO THE

*History or the Art of Medicine, and the Auxiliary Sciences.*

Quicquid agunt medici,
nostri farrago libelli.

NO kind of reading is more universally interesting than Biography. We shall, therefore, fill up a long hiatus, with which we have been charged by some of our readers. The first article is venerable, from the subject, and from his biographer.

*Biographical Account of* Dr. INGENHOUSZ. By the late MAXWELL GARTHSHORE, M.D. F.R.S.

JOHN INGENHOUSZ was born of a mercantile family in Breda, in the year 1730. He was educated at schools there, and afterwards studied at more than one of the Dutch or German universities. He took a degree in physic, and settled in his native town, where he practised with success for a few years. Being naturally indifferent to money, yet strictly economical, and having a moderate independence, he could no longer resist his ardent love of knowledge, and determined, like Pythagoras, Plato, and others, to visit countries and people from whom more might be learned.

From the publications of the Royal Society, and the characters of our philosophers, he was induced to come to London in 1764, and was luckily introduced to Sir John Pringle, through whom he became acquainted with Dr. Franklin, Dr. Huck, and all that circle of literary men that then attended his Sunday and Wednesday nights' meetings. The unassuming mildness and unaffected simplicity of our philosopher's manners, with his unremitting attention to scientific pursuits, soon gained him the favour of all; and his peculiar attention and accommodating respect to Sir John, who was always partial to foreigners, thinking they did more justice to his merit than his own countrymen, rendered Dr. Ingenhousz a particular favourite, and, by degrees, his constant visitor and attendant on all occasions.

Electricity being then a subject of much philosophic discussion, he employed himself assiduously in experiments, and contrived many ingenious methods of improving electrical machines, varying the methods of employing them. He contrived a small machine, which he always carried in his pocket, and with which he used occasionally to amuse his friends. This naturally produced a degree of intimacy with Dr. Franklin, then in high reputation with this country as a philosopher. With him and Sir John Pringle he made one summer a short trip to Paris; and afterwards, with Dr. Franklin alone, a tour through Scotland and Ireland. He continued to pursue his philosophical and medical studies and acquirements in London till the year 1767, when he was recommended by Sir John Pringle to attend the imperial ambassador to Vienna, in compliance with the Empress Maria Theresa's desire, to inoculate her family, in whom the small-pox had been fatal. Dr. Ingenhousz had never before practised, or thought particularly of inoculation, yet such was Sir John's opinion of his sagacity, docility, and general knowledge of physic, and steadiness of character, that, after practising inoculation for some months with Baron Dimsdale, at Hertford, he made no scruple to send him out as fully qualified for that important office, which he successfully executed in the year 1768; and in the year 1769 he inoculated the Grand Duke of Tuscany's family, at Florence. He declined many solicitations to inoculate at the Court of Turin; and, returning straight to Vienna, was appointed Body Physician and Counsellor of State to their Imperial Majesties, with a pension for life. He continued in Vienna for some time, pursuing his philosophical and medical studies. What practice he attended to, I believe, was gratuitous.

He applied himself much to ingenious inventions and experiments; with which he used to amuse the nobility, foreign ministers, and curious strangers, at Vienna, in so striking a way as to gain himself a very high reputation for his *natural magic.*

He some time after obtained leave to travel to France, to gratify his original turn of mind, by witnessing the various experiments and discoveries in pneumatic philosophy, which then began to attract the attention of Europe. He was resident in Paris during the first commotions which took place in the revolution; and was so completely terrified by the outrages then committed, that he conceived, and ever after retained, the most indelible detestation of the principles and practice of the democrats. While at Paris he lived much with Rochfoucault de Maret, and with those other philosophers who were most eminent for their attentibn to, and discoveries in, pneumatic philosophy.

After his success at Vienna, he came to England, in January 1778, and employed himself chiefly in the pursuit of pneumatic philosophy. For this purpose he retired to the country, near London, for some months; and, after a long and laborious course of close investigation, published, in 1779, his experiments on vegetables, demonstrating their power of emitting vital air in sunshine, and azote in the night. These experiments he often repeated, improved, multiplied, and republished, on the continent, with various other philosophical essays, in French, German, and Latin editions. Of the *Experiences sur les Vegetaux*, he published at Paris, in 1787, the first volume, as a much augmented and corrected translation of the English publication of 1779; and, on his second journey to Paris, in 1789, a second volume of the same work, with still more extended views.

On

On his return to Vienna, he married a sister of Professor Jacquin, by whom he had no children, and with whom he did not live many years: his fondness for travelling, and his extreme attachment to England, engrossing the whole of his remaining life. This will be best understood by attending to the chronology and analysis of his numerous publications, which can be noticed in a life of this kind only in a summary way.

The order of his publications, besides those already mentioned in our Transactions, are, first, his Experiments on Vegetables, published in London in 1779, before his first return to Vienna; a translation of which into Dutch was soon after published at the Hague, by Van Breda; and a German translation at Lejpsic, by Molitor, in 1780; each was more enlarged than the original: and, what is remarkable, there were four different editions, published in four different languages, of this work, and all of them in the course of a few months. The first French translation being published soon after he passed through Paris to Vienna, in the year 1780, a second and much enlarged German edition of this work was published at Vienna, in three volumes, in 1786: and a second French edition of the first volume of the same work was published at Paris in 1787; and the second, with many additional improvements, in 1789.

His *Nouvelles Experiences sur divers Objets de Physique* was published in German by M. Molitor, professor at Mayence; and a second part of the same in 1782; and a second edition of the whole was printed in 1784, containing a number of papers already published in our Transactions. This volume begins with a very correct and precise account of the system of electricity by Dr. Franklin; and the second memoir is a very ingenious explanation of all the phenomena of the electrophorus invented by Volta on his theory of positive and negative electricity, the greatest part of which he had already published as a Bakerian Lecture in the Philosophical Transactions for the year 1778. In the third memoir he discusses at great length the much disputed question whether points or balls are the best contrivance to preserve buildings from the effects of lightning. He enters into an examination of Mr. Wilson's experiments performed at the Pantheon, and endeavours to establish the superiority of pointed conductors in opposition to that gentleman, who asserted that they ought to terminate in balls; which last, he says, will rather attract a strong shock; whereas the other, by acting at a much greater distance, will silently and gradually attract and convey to the earth the electrical fire, and so prevent its ever occasioning a severe stroke.

The following memoirs chiefly relate to experiments on inflammable and dephlogisticated air; describing air pistols and lamps; a method of procuring inflammable air from marshes, and in other ways; how to produce the most dazzling light; and how to light a candle at pleasure with an electrical spark; several of which are to be found in our Philosophical Transactions.

In the 13th memoir we have a long account of the nature and best means of obtaining dephlogisticated air from various substances,

 and and especially from nitre. He next republished his paper upon the salubrity of common air at sea compared with the air of the sea-coast and of inland countries, which we have in the Transactions for 1780.* Next a memoir on magnetism and artificial magnets: next a republication of his theory of gunpowder, and of pulvis fulminans. The 18th memoir is on the passage of heat through, and inflammability of, metals; with an attempt to determine the quantity of phlogiston which different metallic and other bodies contain.

Previous to his publication on vegetables, in 1779, he had in the year 1775 published in our Philosophical Transactions, vol. lxv. his experiments on the torpedo, which at that time, from the previous experiments of Mr. Walsh, was exciting much attention; and that subject was further cultivated by experiments on the gymnotus, and by the very accurate anatomical observations of the late eminent Mr. J. Hunter. In the year 1776, Dr. I. published, in vol. lxvi., an easy method of measuring the diminution of bulk taking place in the mixture of common and nitrous air, according to the discovery of Dr. Priestley; and he there describes an instrument he had contrived, whereby this nice experiment might be performed with much more facility and accuracy. In the same paper he published his experiments on platina, which, then a new metal, he had taken much pains to investigate and render fusible. He valued highly a set of buttons of this metal, with which he had a coat mounted. He found platina to be as completely, though not so strongly, magnetic as iron; and that this power was increased by fusion in electrical fire, which he first effected, whilst common fire was found to diminish it. This magnetic virtue he constitutes as a specific property of platina, by which it may be always distinguished from gold, which cannot be rendered magnetic.

In the year 1778, we find a paper in the Phil. Trans. vol. lxviii. describing a ready way to light a candle by a very moderate electrical spark excited positively by a piece of glass, and a match made of cotton powdered with resin. The 48th paper of the same volume contain experiments to explain how far the phenomena of the electrophorus may be accounted for on Dr. Franklin's theory of positive and negative electricity, which he proves to agree perfectly with those exhibited by the late Mr. Canton with elder-pith-balls hanging by linen threads from a wooden box; which balls are excited either negatively or positively by a piece of excited glass.

In vol. lxix. for the year 1779, he gives an account of a new kind of inflammable air, or gas, which can be made in a moment without apparatus, and is as fit for explosion as other inflammable gases in use for that purpose; together with a new theory of gunpowder.

In October of the same year (1779) he published the first edition of Experiments on Vegetables, before mentioned. To this he prefixes a most grateful dedication to Sir John Pringle, explaining at length the whole series of obligations he was under to him for his early patronage on his first arrival in this country, and for his very extraordinary mark of confidence and respect in recommending him to to the imperial family of Germany, leaving this as a public testimony of his gratitude, being then about to return to Vienna.

Vol. lxix. contains, in a Bakerian lecture, a memoir on improvements in electricity, by the use of flat glasses, instead of globes or cylinders, which it now appears he had made use of for 15 years. In vol. lxx. for the year 1780, we find a paper on the degree of salubrity of the common air at sea in the Channel compared with that on the shore, and in various parts of Holland. A letter written from Paris in January 1780, and in vol. lxxii., for the year 1782, contains some further considerations on the influence of the vegetable kingdom on the animal creation.

In the year 1784, two volumes, in octavo, of his various philosophical papers were published in German at Vienna. In 1785, we find *Nouvelles Experiences et Observations sur divers Objets de Physique*, which wholly consist of subjects of electricity, and the different kinds of air, being chiefly what had been before published in our Transactions. This he dedicates to Dr. Franklin, then residing at Paris, as Minister Plenipotentiary from the United States of America. In the year 1789, he published a second volume with the same title, dedicated to Baron Dimsdale, and printed under his own eye at Paris. The second volume contains chiefly experiments and observations relating to vegetables, and especially to that green matter produced in water, on which so much had been said by Dr. Priestley. These two volumes are pretty much the same with those that have been published in German, in which there were also some medical as well as physical papers, which, by a mistake of the editors, were omitted in the French edition.

His last publication, in 1798, the year preceding his death, is an Essay on the Food of Plants and the Renovation of Soils, written at the desire of Sir John Sinclair, and published by the Board of Agriculture, of which Sir J. was president, and of which our philosopher had been made a foreign honorary member. In this paper we have an abridged recapitulation and very ingenious application of his experiments and opinions, so fully illustrated in his *Experiences sur les Vegetaux*, published at Paris, in two volumes, 8vo. in the year 1787 and 1789, to which he continually refers. He surely was the first who demonstrated clearly the singular facts of pure oxygen being continually emitted by vegetables when under the influence of light, by which the air was continually ameliorated, and that of their constantly emitting azote in the dark, by which it is corrupted. It is very true that Dr. Priestley had before him discovered that living plants always ameliorated the atmospheric air, by absorbing phlogiston, a theory in direct opposition to that of our philosopher, who thought this purification was occasioned by their perspiration instead of absorption, which continual absorption of atmospheric air he also allows; and proves, by some most ingenious experiments, that plants derive great part of their nourishment by their leaves; and that respirable air and heat are absolutely necessary to vegetation, though light is not, as they can grow very luxuriantly in the dark, but will emit no oxygen, acquire no green colour, and rather taint taint than ameliorate the air.* As far as concerns the economy of vegetables, he certainly has thrown more light than any other philosopher since the time of Hales, whose ingenuity and success must render him immortal. Though it would require too much of the reader's time to enumerate the variety of ingenious inventions and discoveries which he has published, yet I cannot omit making mention of that very brilliant experiment—the deflagration of solid iron in vital air; of which he was so very fond that he always carried a phial of it in his pocket, in which he used frequently to burn a piece of iron wire, to the great entertainment of his female friends.—*Annals of Philosophy.*

*Account of the Life and Writings of* BARON GUYTON DE MORVEAU, F.R.S. Member of the Institute of France, &c. &c.*

LOUIS BERNARD GUYTON DE MORVEAU was born at Dijon, on the 4th January 1737. His father, Anthony Guyton, professor of civil law in the university of Dijon, was descended from an ancient and respectable family.

Young Guyton's early education was not neglected. But, while his father and his tutor were initiating him in the old routine of theories, Nature taught him to resort to the practical acquisition of knowledge. Accustomed every day to see the various artificers, whom his father employed about his house, to indulge a caprice for building, young Guyton insensibly caught the spirit of mechanics.

A visit which he paid to Voltaire in 1756, at Ferney, seems to have given him at one time, a turn for poetry; particularly of the descriptive and satiric kind. At the age of twenty-four, Guyton had pleaded several important causes at the bar, when the office of advocate-general at the parliament of Dijon was advertised for sale. It is well known that all public situations, even of the greatest responsibility, were then sold in France to the best bidder. Guyton's father having ascertained that this place would be acceptable to his son, purchased it for forty thousand francs. In 1764, he was admitted an honorary member of the Academy of Sciences, Arts, and Belles Lettres of Dijon, then one of the most learned societies of Europe: and here begins that brilliant career, which he followed with so much ardour and glory. A few months after, Guyton delivered an *Eloge* on the President Jeannin, at a public meeting of the Dijon academy: it was full of chaste eloquence, and virtuous sentiments.

The exact sciences were so ill taught, and lamely cultivated, at the time of Guyton's University education, that, after his admission into the academy of Dijon, his notions of mechanical and natural philosophy were scanty and insufficient. He, therefore, applied himself to study the theoretical and practical Chemistry of Macquer; and the Manual of Chemistry, which Beaumé had just published. To the latter chemist he also sent an extensive order for chemical

* Abstracted from Dr. A. B. Granville's Memoirs, in the last Journal of the Royal Institution.

preparations preparations and utensils, with a view of forming a small laboratory next his office. The fresh pursuits of Guyton did not prevent him, however, from continuing to cultivate literature with success. The French academy had proposed for the subject of an *Essai*, the *Eloge* of Charles V. of France, surnamed the *Wise*, and Guyton was one of the candidates.

Chance, which led Guyton to become a chemist, was also favourable in procuring him the means of forming an elaboratory, at a very moderate expense, and with little trouble, by the death of a young chemist at Dijon.

In July of the same year, Guyton went to Paris, for the purpose of visiting the scientific establishments of that metropolis, and of purchasing books, preparations, and instruments, which he still wanted, to enable him to pursue his favourite study. The person to whom he addressed himself for this purpose, was Beaumé, who was then among the first of the French chemists.

Guyton's first essay on his return to Dijon, was presented in a note which he read to the academy that same year, but which, not having been printed, has probably been lost. His next memoir was of more importance. The phenomenon of the increase of weight in metals, after a long exposure to the action of fire, had been, in fact, explained by Jean Rey in 1630; but lost sight of almost immediately afterwards. His explanation was forgotten, and the *calcination* of metals became once more the subject of controversies and conjectures amongst the successive philosophers who cultivated the science of chemistry.

Various other occupations distinguished the next four or five years of Guyton's life, in his double capacity of a chemist and a lawyer. In a dissertation in defence of the theory of phlogiston, then attacked by several eminent men, we find another instance of the accuracy of M. Guyton's mode of making experiments. That theory was ultimately overthrown by the labours of many of our countrymen, and of the illustrious Lavoisier; but the facts relative to combustion, the calcination of metals, and the increase of weight resulting from it, observed and recorded by Guyton in that dissertation, have not only withstood the shock of that mighty fall, but were afterwards admitted by the latter celebrated reformer of chemistry, as some of the best proofs of his new doctrine.

This and the following year of Guyton's life, were particularly rich in useful and ingenious researches. His experiments on adhesion are too well known to need particular mention in this place. The results he had obtained from them were afterwards confirmed by other philosophers, who, like him, found, that a disk of glass, ten lines in diameter, adhered to the surface of mercury with a force equal to a weight of 66.5 grains.

About this time, the theory of gases was beginning to threaten the phlogistic doctrine. Guyton took part in the contest, and perceiving the weak side of the doctrine he had hitherto supported, without, however, being yet wholly convinced of the soundness of that of his opponents, in a publication, which does him infinite credit, and in which his theories and conjectures are supported by new facts and experiments, showed a disposition to adopt the pneumatic doctrine, provided several doubts were explained to him, which yet perplexed and forbade him to embrace its otherwise luminous principles.

[We shall not enter into the controversy concerning the disinfecting powers of gases, or the discoverers of them.]

In 1778, M. Guyton published the first volume of a Course of Chemistry, which shortly afterwards was succeeded by a second, a third, and a fourth volume. The pleasing and desultory part of the science, however, was not the only one that Guyton cultivated. Aware, from his every day increasing knowledge of new and interesting facts, that they were applicable to various objects of public and domestic life, and that they might thus be rendered highly important to society, he studied the different modes of their application; and amongst other enterprises, of which we cannot here undertake to speak, the establishment of a manufacture of nitrate of potash, on a large scale, ought more particularly to be mentioned. This enterprise called forth the thanks of the then minister of finances—the celebrated Necker.

The republic of letters had, in that year, to mourn the loss of two men equally celebrated—Rosseau and Voltaire. Burial service had been refused in Paris to the remains of the latter; and the unprejudiced, as well as the most honest, exclaimed against the proceedings. Guyton, in a discourse pronounced at Dijon, thought it necessary to make an allusion to that event, and to express his indignation, that, in a country like France, men such as Voltaire, whose rights to national gratitude and posthumous fame were countless, should be persecuted even beyond the grave. Although this candid and open avowal of his sentiments exposed, for a moment, the attorney general to the attacks and libels of bigots, yet the approbation of the good and the just was with him; and what other is worth courting?

In 1777 he was charged to examine the quarries of regular schistus and the coal-mines of Burgundy, for which purpose he performed a mineralogical tour through that province. In the memoirs of the Dijon academy for 1779, we find another instance of the useful results of Guyton's new scientific pursuits, in a memoir, giving an account of a rich lead-mine discovered by him, and to work which, for want of other combustibles, he sought for beds of coal in the neighbourhood, though unsuccessfully. A few years later, when Bergman had so well described the properties of the heavy spar and the earth obtained from it, Guyton, greatly assisted by his geological knowledge, searched for it in Burgundy, and found it in considerable quantity at Thôte, so as to be enabled to give to the Dijon academy an accurate description of that mineral, and of the earth which enters into its composition, and which he afterwards called *barote*, or *barytes*.

Known for his writings and his researches, he was requested by Pankouke, who meditated the great project of the *Encyclopedie Methodique*, to undertake the new edition of all the chemical articles cles in that great dictionary, and supported his demand with a letter from Buffon, whose request Guyton could not refuse, though he had long hesitated in accepting the office. The engagement was signed between them in September, 1780; and, although the first part of that work did not appear till six years afterwards, still the study of the researches, which the execution of his engagement demanded, furnished him with immediate and very considerable occupations.

About this period, chemistry derived essential aid from Guyton himself. The nomenclature of that science was obscure and barbarous, and he soon perceived that he should in vain endeavour at perspicuity, while the language remained thus absurd and inefficient. With Guyton, this consideration alone would have checked the ardour of his pursuit, had not his own genius suggested the idea of reforming a nomenclature, till then, the opprobrium of chemistry. To this, therefore, he directed his attention, and, in 1782, was published his first essay on a new chemical nomenclature, forming the basis of all those subsequent changes from which we have derived so substantial benefit, and by the assistance of which, chemistry has grown from a pigmy to a colossus. No sooner, however, did this project reach the capital, than several of the members of the Royal Academy of Sciences, of which he was a corresponding member, inconsiderately undertook to oppose it, and the zealous proposer, as he himself used to say, was *accablé d'objections* in every point of his enterprize. Macquer himself, who, on first learning his friend's project, had written to him, "that, finding it excellent, he had determined to adopt it," did not, in the present instance, dare to defend him against the multiplied attacks of such numerous and powerful opponents. Undismayed, however, and full of zeal, Guyton, with a view to obviate every difficulty, and to answer in person the objections that might be made, went to Paris, and presented himself before the Academy, where he not only succeeded in showing the necessity of the reform, but ultimately induced the most eminent chemists of the capital, such as Lavoisier, Berthollet, and Fourcroy, to join him in rendering that reform more complete and successful.

It would be folly to deny, that the immense progress since made in chemistry—the multiplied discoveries—the luminous mode in which that science is now taught and studied, and its rapid extension, are owing to the zealous exertions of Guyton, as displayed in his plan of a methodical chemical nomenclature, read at the Academy of Sciences of Paris, in 1787. Another circumstance deserving attention is, that from the repeated conferences held with Lavoisier, Berthollet, and Fourcroy, for the purpose of receiving their observations, and corrections of his original proposition, the science derived great advantages: for, while the latter were adopting the ideas of the former, Guyton became convinced of the truth of Lavoisier's new doctrine, and hastened to abjure the phlogistic theory, and to embrace the more luminous tenets of his illustrious countryman.

In 1783, in consequence of the favourable report made by Macquer to government, Guyton obtained permission to establish a manufactory of soda; and in the same year he published his Collections of Pleadings at the Bar, among which we find his "*Discours sur la Bonhomie,*" delivered at the opening of the sessions of Dijon, and with which he took leave of his fellow magistrates, surrendering the insignia of office, having determined to quit the juridical vocations.

On the 25th of April, 1784, Guyton, accompanied by the President Virly, ascended from Dijon in a balloon, which he had himself constructed, and repeated the experiment on the 12th of June following, with a view of ascertaining the possibility of directing those aerostatic machines, by an apparatus of his own contrivance.

At length, the first part of the volume of chemistry of the Encyclopædia made its appearance, and was seized upon with avidity by every one who took an interest in the progress of that science. This work is too well and too generally known, to need any eulogium. The article Acid, alone, is a complete history of the science. The erudition displayed in it, and the numerous and well digested facts to be found throughout—the clear exposition of the various doctrines announced and supported by the different authors who have written on chemistry—and finally, the accurate and full details of the various experiments that had been made in almost every part of Europe, down to the time of Guyton's writings, are in themselves sufficient to ensure him an everlasting fame.

It was Guyton's good fortune to have a man as eminent as himself for a successor in this undertaking. Fourcroy, at the express invitation of Guyton, was applied to by the publisher to continue the chemical part of the Encyclopædia; and, having accepted the proposition, on condition that Guyton should communicate to him his vocabulary—notes—articles already begun—extracts—translations, and drawings; the latter, with that liberality which ever distinguished him, lost not a moment in sending him every thing he had asked for, together with the entire article on Metallurgy by Duhamel, and the plates relating to it.

In September 1787, our professor was pleasingly surprized at Dijon, by the honour of a visit from Lavoisier, Berthollet, and Fourcroy, accompanied by their ladies, and MM. Monge and Vandermonde. By a very lucky coincidence, Dr. Beddoes, of Bristol, who was travelling through France at the time, happened to pass through Dijon, and joined this party, who had assembled to repeat and discuss several experiments explanatory of the new doctrine.

In 1790, Guyton received a letter from Count St. Priest, naming him one of a commission, appointed by the Assembly, for the formation of the department of the Côte d'Or. While thus engaged for the public service, he received a fresh mark of the high consideration in which his talents and knowledge were held by the most eminent among the learned of his nation; and on the 25th of August, 1791, obtained, from the Academy of Sciences, the great prize, which was annually decreed to the most useful work, for the first volume of the Dictionary of Chemistry in the Encyclopædia.

[We

[We shall pass over the political events of this life, which, though interesting, are well known.]

In 1795, Guyton was re-elected member of the Council of Five Hundred, by the electoral assemblies of Sarthe and Ile et Vilaine; when, the executive government having decreed the formation of the National Institute, he received a letter from the minister of the interior, announcing that he had been named one of the forty-eight members chosen by government to form the nucleus of that scientific body. In 1797 he once more attached himself exclusively to science and to the establishments for public instruction. The absence of Monge, who was then in Egypt, requiring the appointment of a provisional director of the Polytechnic School, Guyton was appointed to that responsible situation by the Directory, in 1798, and continued to exercise its duties during nineteen months, to the complete satisfaction of every person connected with that establishment.

At the end of 1799, Bonaparte, as first consul, appointed Guyton one of the *administrateurs generals* of the mint; and the year following, director of the *Ecole Polytechnique*.

The legion of honour had been but recently instituted for the reward of eminent services rendered to the state, when Guyton received the cross of that order from the hands of the first consul, in the church of the Invalids. His promotion to an officer of that same order, took place in 1805, two years only after obtaining his first decoration.

Nor did the patriotic and enlightened imperial government stop here in its public demonstrations of that consideration and esteem which Guyton so fully deserved, for, in 1811, he was created baron of the empire, a title recognized by the succeeding administration.

Sixteen years of uninterrupted labours at the *Ecole Polytechnique*, since 1798, (for, notwithstanding all his other occupations and responsible situations, he had not, for a single moment, ceased from his duties as professor of chemistry at that school) seemed to entitle him to an honourable retreat. This he obtained on application to the proper authorities, and withdrew from public into the retired station of private life, crowned with years and reputation, and followed with the blessings of the numerous pupils whom he had brought up in the career of science:—but alas! for them, and his numerous friends, his time of repose in this world was to be but short: he lived but three years more to witness still greater changes in the politics of his country, than those to which he had been instrumental, and to see the complete overthrow of that glorious system of liberty which had been supported by the majority of the best thinking men in France. Guyton was seized with a total exhaustion of strength on the 21st of December, 1815, and expired in the arms of his disconsolate wife and a few trusty friends, after three days' illness, having scarcely completed the eightieth year of his age. His remains were followed to the grave by the members of the Institute, and many other distinguished characters of the capital, on the 3d of January, where Berthollet, one of his earliest colleagues, pronounced, pronounced, according to custom, a short, but impressive funeral oration on his departed friend, which has not been allowed to be printed in France, because Guyton, in honestly voting for the death of the king, had acquired the name of regicide.

"No one (says Dr. Granville) ever exerted himself with greater assiduity, during a very long series of years, in public and private life, than Guyton de Morveau.—*Procureur-général* for the space of two-and-twenty years at Dijon—solicitor-general of one of the French departments—member of the legislative body—of the national convention—of the council of five hundred—occasionally upon the committees on financial, diplomatic, and legislative subjects—commissary to the armies and to the frontiers—and, finally, director-general of the mint, he might fairly be supposed to have had no time for any other occupation. But, when we reflect, that he was still better known for his services to science—that he was a public professor of chemistry at Dijon and Paris, for upwards of thirty years—that he fulfilled the duties of director-general of the Polytechnic School for a considerable space of time—and, finally, that during twenty-six years, as member of the National Institute, he furnished several important memoirs and reports to that scientific body, published in its transactions, besides many other papers printed in the *Annales de Chimie*, of which he was one of the earliest and most indefatigable editors—we cannot withhold that tribute of praise and admiration which his memory demands."

*Account of* Dr. WILLIAM SAUNDERS, *Fellow of the Royal College of Physicians, Physician to his Royal Highness the Prince Regent, and late Senior Physician to Guy's Hospital.* (From the Picture of the College of Physicians—see our Review.)

To contemplate the labours of a well-earned life, in the evening of retirement, is the enviable satisfaction of few, though to be desired by all: and the respectable individual, the subject of this memoir, can, in his quiet retreat in the country, look back with the calmness of philosophy on the bustling scene he has left, and observe the conduct of those who are pursuing the same track, and climbing, with various success, the steep and too often craggy path, that leads to reputation and fortune in medicine.

Dr. W. Saunders is a native of the North of Scotland, and in the neighbourhood of his nativity he received the rudiments of classical learning, and those other branches preparatory to professional studies. The department of medicine being his favourite pursuit, at a fit age he was sent to the University of Edinburgh, where the celebrated Dr. Cullen then filled an academic chair. It was the peculiar anxiety of this distinguished character, with a view to spread his opinions, to select those pupils as his favourites who were most marked for their abilities, their industry, and popularity. Dr. Saunders had the good fortune to be of this selection—a proof of the high opinion thus early entertained of his merit; and to live with him in the most intimate footing, as an associate and friend.

4 Under

Under so eminent a tutor, and so particularly sharing his esteem, Dr. Saunders could not fail to make a rapid progress in his profession. He accordingly took his degree in 1766, as Doctor in Medicine, with much credit; and published a Dissertation on Antimony, shewing great chemical knowledge and research. Thus finished in his professional attainments, he looked forward to the capital as the land of promise, and a settlement here was the first object of his ambition. He accordingly commenced practice, and was equally fortunate in his outset, by forming a similar connexion with Sir G. Baker, President of the College, as he had done with his former tutor. Sir George was then engaged in his celebrated Dissertation on the Devonshire Colic, the source of which he referred to an impregnation of lead. To establish this fact, the chemical talents of Dr. Saunders were called into action, and a variety of nice and complicated experiments were conducted by him with this view, so greatly to the satisfaction of Sir George, that, as a spontaneous act of gratitude, and from the strong impression of Dr. Saunders's rising abilities, he proposed him to the College, to have all the honours and privileges of a Fellow, without having studied at any English university—a concession only granted to high merit and correct conduct. This certainly was a most creditable introduction to Dr. Saunders, and gave him an opportunity of shewing his classical acquirements, by delivering the annual Harveian oration, which he afterwards printed.

Soon after, he was also equally successful, by a vacancy occurring in Guy's Hospital, to gain the appointment of Physician to this important and extensive medical establishment. Here a field immediately opened for properly displaying the energies of his mind, and his professional acquirements; and his industry, exertion, and talents, did not lose sight of the opportunity put in his power, of standing high in scientific reputation, and reaching the acmé of medical respectability. The situation of Guy's Hospital is particularly favourable to early initiating its medical attendants into the emoluments of extensive practice: the numerous and respectable circle of its governors, and the large pupillage it has always commanded, render a popular physician there on an eminence, to be seen, talked of, and to have his merits fully discussed. Of this ordeal Dr. Saunders's abilities were fully competent to stand the trial, both as a teacher, a practitioner, and a gentleman of general and varied information; and the consequence accordingly was, that under his direction the reputation of the Hospital, as a medical school, first commenced, and gained its high character: he stood himself at the head of the city practice in a few years; and his countenance and support was courted in every liberal and scientific undertaking that went on, either as a party, an adviser, or friend.

Possessed of much good sense, prudence, and a just discrimination of character, the connexions formed by Dr. Saunders were valuable and important; and the esteem in which he was held by them enabled him to use them for the best of purposes, and to deserve a reputation above what medical abilities can bestow—the merit merit of being the patron of the young and unbefriended of the profession.

A concurrence of circumstances favoured his benevolent inclinations in this respect. At the breaking out of the late war, so long and disastrous, several of his pupils possessed high departments in the Army and Navy Boards, and their respect to their former preceptor made them pay every attention to his recommendation. In one year four army physicians owed their preferment solely to his interest.

His influence with the Directors at the East-India House enabled him to do the same to a considerable extent; and, after performing the part of an enlightened instructor and able lecturer, in conveying professional information, he ushered his pupils into life with the advantage of certainty and preferment. The consequence of this was, that no one was better acquainted with the professional improvements going on in every quarter of the globe than this gentleman. His good deeds of service and patronage formed ramifications of friendship and correspondence from the most distant regions, which returned their streams of intelligence, as marks of gratitude, to their parent benefactor. His lectures, therefore, which were highly popular and well delivered, were furnished with every thing new in respect to information on disease, and he had the earliest opportunities of giving a trial to every novel remedy, that either had been suggested, or put to a trial by others.

Thus, he was the first who proposed to Dr. Percival, in a letter published by that eminent physician, the use of the carbonic acid in calculous complaints; and in the pharmaceutical treatment of mercury he introduced an important improvement, that of making a mercurial ointment with more certainty and less labour, by a new preparation of its oxyde, in adding lime-water to calomel, and with this oxyde forming the ointment—an ointment far more readily absorbed, and possessing more activity, than that commonly prepared by triture.

Though thus immersed in an increasing routine of extensive practice and varied engagements, such was the order of Dr. Saunders's mind, and such his regard for the pursuits of literature, as not to overlook the character of the author as well as the physician.

His first publication was, a Treatise on the Red Bark, in which he has stated the particular qualities of this species compared with the others, and in which he has offered a number of practical remarks on the use of the bark in general.

This treatise was well received; and, from the authority of its author, gave a preference for a time in practice to that species of this valuable remedy which was the subject of it. This work was followed by another and more important one, a Treatise on the Liver and its Diseases, which may be considered as his standard production. It was first read before the College as the subject of the Gulstonean lecture; and, in consequence of being so well received by that learned body, he was induced to give it to the world in its present shape. He was also naturally led to the subject from his his East-India connexion, and the number of Anglo-Asiatics who, on their return, put themselves under his care. He has there laid the foundation of the *Hepatic Theory*, now so universally adopted by his successors, but without that judgment, on their part, he has displayed in the use of the specific so much recommended in Asiatic practice. His earnestness to enforce a proper and regulated use of this means led him lately, in a small pamphlet, to resist the late opinions of others, and to oppose the solemn admonitions of practical experience to the jejune and immature notions of fanciful hypothesis.

From this acceptable production to the profession and the public, he was next induced to turn his attention to another of no less importance, and which united chemical abilities with practical knowledge, and clinical observation in treating it. This is his work on Mineral Waters: a work of high merit and utility; and which shews every one, when at a watering-place, what he should prefer, and what he should avoid, during his residence at these seats of health and amusement. This production was much wanted. The former treatises on mineral waters were either obsolete or incomplete, and this has supplied the desideratum which every patient looked for.

Dr. Saunders's elevated character as a physician gained him a ready admission into the first and most distinguished literary associations. He was elected early a Fellow of the Royal Society. He was President of the New Medical Society, founded on a select plan; and he belongs to most of the public institutions of consequence in the country.

From his extensive information, and standing at the head of the city medical department, no one had it more in his power to benefit the profession in general than Dr. Saunders. He took, at this period, a delight in knowing whatever was going on in medicine in the different parts of the country; and that knowledge he applied to serve his friends, and in pointing out where they could best settle, and where he could most serve them by his introductions.

Such a character could not fail but to be generally respected by the profession; and the friendship that subsists between him and many of the first individuals of this class, is a strong proof of it. The intimacy between him and Sir W. Farquhar has continued for upwards of forty years, with mutual advantage to both; and, in consequence of that friendship, Dr. Saunders had the honour of being appointed Physician to the Prince Regent, and was also called in to attend the late Princess Amelia, on the supposition that her disease was connected with an affection of the liver.

Dr. Saunders's patronage of Dr. Babington does him no less credit than the other parts of his conduct. This gentleman, who has succeeded him in this hospital, and in his city practice, owes his rise in life entirely to his introduction. Commencing in what may be termed the initiatory department of the profession, he gradually rose in medical honours and preferment, till the resignation of Dr. Saunders in his favour, on the latter's retiring to Russel-square, and quitting quitting his city connexion. Many other acts of similar benevolence and philanthropy might be instanced.

Thus, in concluding the character of this respectable individual, we may justly say, his earlier days were marked by much industry and exertion, in establishing his professional reputation; in the zenith of his practice and fame, his influence and connexions were employed to the best and most benevolent ends; and in his present retirement he carries with him the regrets of the profession, to the young of which he was always accessible, and to them his advice and services were always extended. Though, as we have noticed, he is properly the founder of the modern Herpetic System of Medicine, he may be truly said to take no impression from his hypothesis, or to view the world with *a jaundiced eye.*

The restoration of Dr. Saunders's health, and his return to the capital, is anxiously wished for, especially by the junior part of the profession, who cannot fail, in consultation with one of his long experience and enlarged observation, to profit greatly by his opinions as a consulting physician.

*** There are a few errors in the above, which we make no scruple to correct. But, first, it is worth remarking, that we have no date to any event excepting the taking a doctor's degree in 1766. After this, it appears by the account as if Dr. Saunders had been chosen a Fellow almost as soon as he appeared in town, and before his appointment to Guy's Hospital. This ought to be corrected, because it is impossible to say how far such an error may mislead those who are ambitious of collegiate honours. Dr. Saunders was chosen physician to Guy's on the first vacancy that occurred after his arrival in London: but it was not till some time after the year 1790 that he was admitted to the honour of a Fellowship in the London College. For this distinction he was supposed to have been much indebted to his countryman Dr. William Pitcairn, at that time President.

Dr. S. never professed to be a profound classical scholar; he was a man of quick genius, amiable temper, and very warm and sincere in his patronage and assistance whenever he undertook either. In the latter part of his life he was much affected by some apparently arterial irregularity about the brain, which, though it never impaired his just conceptions, rendered application extremely painful to him. He recovered in great measure from this, and his friends again indulged a hope of sharing his convivialities, his friendship, and his advice. By the date of his degree, he must, however, have exceeded his 70th year some time before his death.—EDIT.

DR. C. COMBE, *Licentiate in Midwifery of the Royal College of Physicians, and Physician to the British Lying-in Hospital.* (From the same.)

Besides great professional and classical knowledge, this gentleman has distinguished himself by subjects of science, not so often cultivated by the professional character, the love of historical and and antique research, in both of which pursuits he stands deservedly high.

Dr. Charles Combe is a native of London: his father was an apothecary in considerable business, for many years, in Southampton-street, Bloomsbury. The doctor received his classical education at Harrow, where, having risen to the sixth form, he left school between 16 and 17 years of age, and was to have been entered at Queen's College, Oxford, but his elder brother, who was then with his father, being in a bad state of health, and soon after dying, the doctor continued with his father, who procured for him every assistance in his power for the prosecution of his studies.

At the age of 19, he became a perpetual pupil to Mr. Moffat, surgeon to the Middlesex Hospital: during the time he attended Mr. Moffat, he regularly dissected the subjects for lectures, two or three years; he also attended the different professors in medicine, chemistry, and natural philosophy.

In January, 1771, he was elected a Fellow of the Society of Antiquaries, and in January, 1776, he was elected a Fellow of the Royal Society, into both which his known acquirements gave him easy access.

Having thus traced Dr. Coombe's professional progress, we must not omit noticing what he has contributed to our fund of general and professional information. In the year 1780, he published a Description, in 4to. of the large Brass Coins of the 12 first Cæsars: this work, which was dedicated to the Marquis of Rockingham, evinced an intimate acquaintance with medalic history. This science of medals has been too little cultivated in this country, as, in addition to many other advantages, medals possess the very important one of recording events which historians have passed over in silence, and which they alone have not failed to perpetuate.

In 1782 Dr. C. published a Catalogue, in 4to. on an entire new plan, of the Coins of the Autonomous Greek Cities, in Dr. Hunter's Museum, illustrated by an extensive and well-executed series of plates, in which the coins are represented of their true size, and with the most scrupulous fidelity,

Dr. William Hunter, at his death in March, 1783, left him, jointly with Dr. George Fordyce and Dr. David Pitcairn, executor and trustee to his museum. The confidence thus reposed in him is, perhaps, the most honourable testimony of friendship that could be shewn, and an evidence, at the same time, how competent Dr. H. considered him to the task of a trust which required not merely correct and conscientious conduct, but also the proper fund of science to estimate its value.

In 1784 he received the degree of Doctor of Physic from the University of Glasgow: in the same year he was admitted a Member of the College of Physicians; and, the year after, was elected a governor of St. Bartholomew's Hospital, on the recommendation of Dr. William Pitcairn, President of the College of Physicians, and one of his Examiners,—a proof of the high opinion entertained of him by that distinguished character.

In February, 1789, he was elected Physician in Ordinary to the British Lying-in Hospital, Brownlow-street, which situation he resigned February 16, 1810. As a return for the very zealous and honourable discharge of his duties there, on the next Special General Court, he was unanimously elected Consulting Physician, and afterwards received the following note from the secretary.

*April* 13, 1810.

At a Special General Court, the Hon. Philip Pusey in the chair—

Resolved, That the unanimous thanks of this Court be returned to Dr. Combe, for his long and faithful services as one of the Physicians in Ordinary to this Hospital.

In addition to the proofs of Numismatic learning, Dr. Combe, in 1793, edited, conjointly with the late Henry Homer, of Emmanuel College, Cambridge, a Variorum edition of Horace, in two volumes quarto: a work which will not be easily superseded, either with respect to the judicious selection of the notes, or the elegance of the typography.

Besides these works, Dr. Combe has, at different times, sent various small essays, principally on subjects connected with his profession, and mostly without a name, to different periodical publications.

In 1783 he published, in Mr. Maty's Review, a critical Examination of the (then new) Edinburgh Pharmacopœia, in which he shews ability as a chemical physician and pharmacopolist, and points out several imperfections in the work which was the subject of his criticism.

In 1814 he sent to the College the case of W. P. G. Esq. which they did him the honour of publishing in the 4th volume of their Transactions. This is a singular case of stricture and thickening of the ilium, occasioning an uncommon pulsation of the aorta, not depending on any diseased structure of the artery. The pulsation had existed for several years, and was not only perceptible to the patient internally, but even by the hand applied externally upon the umbilical region.

In closing our account of this gentleman, it may not be improper to notice, that he possesses a very extensive medical library, and more particularly in the class of obstetrical books, perhaps the most complete private collection in this or any other country. We feel great pleasure in adding, that no man has a greater claim to the character of the finished scholar and intelligent physician; and that he has, consequently, enjoyed through life that distinguished connection with the learned which gives him high respectability in the College list.

*** Such is the brief account of one of those valuable men who serve their generation with the greatest steadiness, but often, for want of starting properly, if we may use such a term, or of finding the proper road afterwards, are nearly neglected at the close of life, and and forgotten soon after death. Dr. Combe was an able practitioner, a profound scholar, considering that he had another department which he never neglected, and, what is most to the purpose, a most amiable and candid man. By the history of his life, the materials for which, we presume, were furnished by himself, it appears that, though, in the year 1771, he was thought worthy of a seat with the Society of Antiquaries, and, five years after, was honoured with the distinction of F.R.S.; yet, that he had not a degree in physic till 1784, when he probably owed the title to his executorship and trusteeship of Dr. W. Hunter's Museum, which, it is well known, was, by the Doctor's testament, to be removed to the University of Glasgow at the expiration of thirty years after his death.

By the above account it seems to follow that Dr. Combe continued the practice of pharmacy till after his 40th year, with a business prepared to his hands, and with an education and means of improvement far greater than most of his competitors. The drudgery of pharmacy was certainly ill suited to such acquirements; and it is not improbable that the world considered him rather as a scholar and a numismatist than as a physician. At the same time, other professed scholars might not be as willing as the meek Mr. Henry Homer to admit a physician to all the honours, which are generally the more esteemed as they are the more rare. Hence a trifling inaccuracy in that part of the edition of Horace which was printed after the death of Henry Homer gave the critics an opportunity of exulting, which was at length re-echoed with tenfold force in the "*Pursuits of Literature.*"

Whatever may have been the cause, it is certain this truly valuable man died poor, his books being sold for the benefit of his creditors, and sold in a manner which added a new pang to those who felt for the deceased, and for medical science. It redounds much to his credit that, under all his difficulties, he educated his children in such a way as to render them ornaments to that circle in which they move. Mr. Taylor Combe, by the employments he has filled, and the works he has published at so early a period of life, already reflects on the memory of his parent that honour to which he was justly entitled during life.

### Dr. Wells, *Licentiate of the Royal College of Physicians.*
(From the same.)

Even in the most aristocratical bodies, we find the spirit of freedom burst forth at times beyond restraint, whenever restriction is carried too far. The laws of the College are certainly too rigid; but the administration is generally conducted with prudence, and without regard to their letter. Frequent, however, have been the disputes between the Fellows and Licentiates, respecting their rights; and on the last of these memorable occasions, the subject of the present memoir shewed himself the advocate of his own and his brethren's cause.

Dr. Wells is a native of America, of Scots extraction, but was educated chiefly in this country. Edinburgh was the university at which he acquired his professional learning, and took his degree. He became a Licentiate of the College, and then settled in the metropolis, where he has since continued to practise. With an independence above his wants, and naturally of a philosophic turn of mind, he has never shewn himself over anxious in the pursuit of business.

Though mild and inoffensive in his manner and character, what, perhaps, he would have overlooked in his own person, he stepped forth to vindicate in the cause of another; and his publication in favour of Dr. Stanger does credit to his pen, and to the cause in which it was employed.

The restrictions of the College, at this period, on the Licentiates, were uncommonly severe, and not suited to that liberality which should distinguish a learned body. Dr. Stanger's application to the College being refused, an application was made on the subject of its powers to the Court of King's Bench. A committee of Licentiates was appointed to manage it, of which Dr. Wells was one, in conjunction with Dr. Cooke and Dr. Stanger; and the subject was ably handled by him, in a publication, which, for sound argument, liberal sentiments, and correct style, does him high credit. The business was ended by concessions on the part of the College, which have now set it fully at rest. Since that time, Dr. Wells has not particularly pressed himself on public notice, fond of that literary retirement, and those scientific pursuits, so pleasing to a cultivated mind.

On the vacancy of Physician to the Honourable East India Company, by the death of the late Dr. Hunter, he stood a candidate; but was out-voted by Dr. Dick, from a principle then laid down by the Company, that this appointment should be filled by one of their own servants, as more conversant with that particular line of practice it was fit their surgeons should be made acquainted with. This, however, was taking the office out of the regular channel in which it had hitherto flowed, and considered rather as a reflection on the College at the time.

Dr. Wells has published several papers in the Phil. Trans. on philosophical subjects, and lately a work upon Dew. He is also the author of several useful papers on medicine, to be found in the Transactions of a Society for the Promotion of Medical and Chirurgical Knowledge.

*** Dr. W. was one of the refugees from America in consequence of the revolution. His talents are too well known to require any encomium. His love of order, and accuracy in tracing facts, shone in all his philosophical inquiries; but in none more than his treatises on Vision, and his researches into the causes of Dew. It is not a little remarkable that one so much attached to the existing order of things as to give up every thing for maintaining royalty, should be the most zealous in resisting the long-established privileges of the

Royal

Royal College of Physicians. It certainly very much resembles what is often said, that the only wish of zealots is to acquire those emoluments they see others possess, or to extinguish them altogether. It is hardly possible to believe, that, had Dr. W. been educated at Oxford or Cambridge, he would not have been as devoted to the privileges of his Alma Mater, as he proved to the royal cause. These remarks will be rather considered as directed to the human character than to Dr. Wells, the goodness of whose intentions are universally admitted. They may, however, teach us all a little moderation in the opinions we are apt too hastily to form of others. They may teach us, before we engage in any warm opposition, to place ourselves in the situation of those we oppose; and it would be well also if the party in power would reflect with how much more ease they would retain that power if tempered with a liberality by which they might reign without seeming to reign. In a word, if all men would endeavour to soften the intercourse with each other, and to do unto others as they wish they should do unto them.

### *Biographical Account of* ABRAHAM GOTTLOB WERNER, late Professor of Mineralogy at Frieberg.

Abraham Gottlob Werner was born on the 25th of September, 1750. His father, who was inspector of an iron-work at Wehrau, on the Queiss, in Upper Lusatia, intended him from his early youth for a similar vocation. Even while at the university, he employed himself on the doctrine of the external characteristics of fossils, in which a singular quickness of perception was of great use to him; and published there, in the year 1774, the well-known work (on the external characteristics of fossils) which is still considered as the basis of his whole oryktognosis, but of which he could never be induced to print a new and enlarged edition, because he feared disputes, and had not in fact concluded his researches. Soon after he was invited to Frieberg, to have the care of the cabinet of natural history there, and to read lectures upon it. Here his mind, which was early exercised in observation and classification, found the most welcome materials. Here, daily extending the bounds of his science, and supporting its foundation by the surest external distinctive marks, he formed that system which,—afterwards embracing also the geognosis which was peculiarly his own, and forming an intimate connexion with all branches of the art of mining,—gradually surmounted all opposition, and raised its inventor to the rank of the creator of a new mineralogy, which might be supported and extended, but not rendered useless, by the crystallographic theory of Haüy, and the chemical theory of Vauquelin and others.

His peculiar talent for observation was animated by the most lively fancy, assisted by the most extensive reading in every branch of knowledge connected with his own, and excited by daily intercourse with ingenious travellers and foreigners, who chiefly visited Frieberg on Werner's account. (We may instance only the Englishman Hawkins.) The classification in genera and species, and for the most part ingenious appellations of minerals down to the newest *egron*, is peculiarly his. "Werner," says Leonhard, in his eloquent lecture on the state of mineralogy, "was for the doctrine of the recognition of simple fossils, embracing with uncommon ingenuity all the experience of his age, what Winckelmann had been to the arts. What, before him, were all the endeavours of Wallerius and Linnæus!" How soon was he obliged to give up Cronstedt, who is no where satisfactory! Only a too scrupulous conscience prevented him from publishing the oryktognostical [fossilological] tables, which have been finished, and quite ready for the press these four years. Werner sustained an obstinate, but for that reason the more honourable contest with the volcanists. Now, no well-informed person will consider the basalt and other fleetz mountains as of volcanic origin. Werner's theory of the older and newer formation of mountains, by the waters, stands immoveable; and a satisfactory link between them is afforded in the mountains of the interval of transition. Even the new chemical discoveries of the *kalimetals* may be made to accord with it. Another science, Mining, on which Werner used also to lecture, was rendered extremely clear to the attentive scholar, by his luminous explanation, and by the reduction of the most complicated machinery to the most simple propositions, at the same time drawing all the figures on his table. Indefatigable application, insatiable thirst of knowledge, enriched his retentive memory with every thing that history and philology, in the most extensive sense, can offer to the attentive inquirer. No science was foreign to him, but all served as a basis to his studies, which were constantly directed to natural philosophy, and the knowledge of the earth and its inhabitants. He always advanced before his age, and often *knew* what others only *presumed*.

After 1779 and 1780, when he first lectured on oryktognosis and geognosis, at Frieberg, he was heard with gratitude by scholars from all parts of Europe. Never contented with what was discovered, always seeking something new, he rather formed scholars who wrote than wrote himself. Many manuscripts, almost wholly ready for the press, are included in his fine library, with a collection of coins and manuscripts bequeathed on the day of his death to the Mineralogical Academy, for 5000 crowns.

In his lectures he had only heads of the subject before him, and used to abandon himself, as he was accustomed to say, to the inspiration of his mineralogical muse; and, when his spirit hovered over the waters and the strata, he often became animated with lofty enthusiasm. But he caused his lectures to be written out by approved scholars; and by revising himself what they had thus written after him, made it, properly speaking, a manuscript. A great many parts of his lectures have been made public by others, among which may be reckoned what Andic, at Brunn in Moravia, has published in the valuable journal Hesperus. But nothing bears the confirmation of the seal of the master. What is particularly desirable is the publication of his manuscript on Mineralogical Geography (which he only once drew up for a particular lecture), and upon the Literature of Mineralogy; ralogy, in which he solved the difficulties of the ancient classic mineralogy, and gave incomparable illustrations of Pliny's Natural History. He was like a father to all his scholars, to whom he was a model, not only as a man of science, but as a moral character. Having filled, from the year 1792, a high situation in the Council of the Mines, he had a great share in the direction both of the Mineralogical Academy and of the administration in general.

Two things must be mentioned here with particular honour—the works begun in 1786, to furnish a great part of the deeper mines with water, in order to get water for driving the wheels. This astonishing aqueduct, particularly the artificial canal of Doerrenthal, with its subterraneous bricked channels, already extending above a league, are in the main due to him, though Scheuchler made the plan, and Lampe the calculations. By the continued support of the ever active King of Saxony, this great work still proceeds in the most prosperous manner. The Amalgamation works, twice built by the excellent Charpentier, chief of the Council of the Mines, (the first building was maliciously burnt down,) and for ever secured by most ingenious fire-engines from similar accidents, are indeed unique:—a miracle to all who behold them, and a jewel in the crown of the Saxon art of mining, and of the unostentatious energy with which the sovereign of Saxony caused the most expensive undertakings to be executed in silence. Less known and visited by foreigners, though on it depends the continuation of the mining in Saxony, is this undertaking of canals and aqueducts, which has already cost above half a million of crowns, and on which more than a thousand men are employed. The mineralogical survey and description of all Saxony, divided into districts, which has been prosecuted for these twenty years, under scholars of Werner, and includes the forest of Thuringen, and even a part of the Harz, uniting too with the mountains on the frontiers of Bohemia and Silesia, will one day give our country a mineralogical map, which for exactness and extent surpasses what any other country can produce. This too was Werner's work, and was constantly directed by him in the most attentive manner. In his visits to Prague and Vienna, he found means to interest the Austrian government in these mineralogical surveys; and it is to be hoped that the enlightened Bavarian government, as well as the direction of the mines in the Prussian monarchy under Werner's grateful scholars in Berlin and Silesia, will readily contribute to support and complete the great work which Werner so happily set on foot. In England and Scotland excellent mineralogical maps of single counties have lately been published according to Werner's ideas.

His cabinet of minerals, unrivalled in completeness and scientific arrangement, and consisting of above 100,000 specimens, has become, in consideration of a life annuity, the amount of which devolves to the institution itself, the property of the Frieberg Mineralogical Academy. Werner's favourite pupil, Koehler, is appointed inspector of it. Werner had received from England an offer of 50,000 crowns for it. He sold it to his country for 40,000, of which he reserved the interest of 33,000 as an annuity; but made the condition, condition, that, after his own death, and that of his only sister, who is without children, the interest should continue to be annually paid to the Mineralogical Academy; so that this, his only daughter, as it may be called, obtains an additional annual income of 1600 crowns.

Werner's literary studies, like his mind, embraced every branch of science. Every thing excited his thirst of knowledge, and thus it often happened that he dedicated all his attention to researches which seemed to lie entirely out of his sphere. His inquiries into the direction of the mountains of the first and second formation, led him to the seat and the migrations of the aboriginal tribes and their branches. To this were soon joined inquiries into their original languages and radical syllables, which he prosecuted with the greatest acuteness, and reduced into tables. Soon arose an universal glossary of all the radical syllables and characteristic sounds, in all the languages with which he was acquainted; which he studied with ardour, and to complete his knowledge of which, he purchased the most expensive works; thus he gave sixty crowns for Hickes' Thesaurus, and but lately eighty crowns for Walton's great Polyglot. His antiquarian researches into the mineralogy of the ancients made him a passionate friend of archæology, and the most costly works on that subject were purchased by him. One branch of archæology, the numismatology of the ancients, had become so favourite a pursuit with him during the last eight years of his life, that he purchased entire collections of medals, and in a short time was in possession of above 6000 ancient Greek and Roman coins. This enabled him to make interesting researches into the different mixtures of the metals, and on the arts of adulteration; and in order to make all more clear, he arranged entire series of false coins. An unedited silver coin of his collection, which he gave to the great connoisseur Catauro, in Milan, is still the subject of a numismatic controversy between the Vienna and Italian connoisseurs. The examination, which was to be printed, was intended to be dedicated to Werner. The practice which he had of studying the direction of the mountains and the surface of the earth, made him an excellent judge of ground, and inspired him with a great fondness for military tactics. He studied the art of war with great diligence, read the accounts given by masters in this branch, and acquired a fine collection of military books. Officers of the engineers and general staff were surprised to hear him speak of the mistakes committed by the allies from want of due knowledge of the ground, in their attack upon Dresden in August 1813, where he happened to be present. His name was mentioned at the head-quarters of the allied sovereigns at Frankfort, and he was invited to repair thither; but his inflexible attachment to his king made him decline the invitation. Medicine also attracted his attention, at first as lying in the circle of the sciences connected with natural history, but afterwards in the latter years of his life, that he might be enabled to judge of the bodily sufferings of himself and others; so that medical books were his favourite reading, and conversation on medical subjects what he preferred to every other. Ever ready to afford assistance, he was happy, when he visited a sick friend, to be able to give medical advice, and also to judge of his own situation which he often thought precarious. The danger of such an inclination, which can never lead to any thing further than empiricism, is evident. His best friends, among whom we may reckon the veteran of the healing art, the venerable Dr. Kapp, at Dresden, sometimes reproved him for this; but it remained his favourite hobby-horse. He had made a very witty table of diseases according to the stages of human life, from infancy to old age; he was a sworn enemy to vinegar and all kinds of milk diet, but a determined beef-eater. In other respects he lived very temperately, drank but little wine, and was especially and anxiously careful about warm clothing and warm rooms.

He first visited Carlsbad, when only fourteen years of age, and had since been there forty-one times. Here, even in the latest part of the autumn, he always acquired new strength. Had not imperious circumstances hindered him this time from visiting sooner the salutary fountain, which had become absolutely necessary to him, he would perhaps have still lived. He was fond of travelling, and spoke with emotion and pleasure of his visit to Paris in 1802, where he was received with the greatest respect. Though not indifferent to external distinctions, to the diplomas of foreign academies and learned societies, he never sought or asked for them, and in conversation never attached any value to them. However, he was justly proud of being a member of the Institute of France, and of the Wernerian Society in England. Even on his death-bed he learnt with joy from his former pupil and faithful friend, the Professor of Natural History at Edinburgh (Jamieson), that not only several mineralogical societies flourished in Great Britain, but that professorships of mineralogy on Werner's principles were founded at Oxford, Cambridge, London, Glasgow, Cork, Dublin, and Belfast. At his suggestion an union of friends of natural philosophy and mineralogy was formed last winter in Dresden, where Werner himself presided.

He was in the best sense of the expression a citizen of the world. Every newspaper that he read, excited in him a pious wish for the happiness of mankind, for truth and justice. In the last days of his life, his eye was most frequently directed to the Brasils, where the excellent Oranjo was his friend, and many Germans now employed there his scholars. In his thoughts he followed every traveller, and put questions to him, in his own mind, such as Michaelis once wrote for Niebuhr and Forskael. His house was the constant rendezvous of curious travellers, from all countries and of all ranks; and he showed to them all, with uncommon patience and attention, his museum, and especially his collection of precious stones, which excites surprize by the value and variety of the specimens. He did not, however, like writing letters, because he preferred personal intercourse to every thing, and dreaded a loss of time. This disinterested participation, in whatever promoted in any country the interests of knowledge and humanity, did not hinder him from being the most faithful son of his own country, the most loyal reverer of his king. He refused every invitation from abroad, (and he received ceived at an early period several very brilliant and enticing ones,) and was for many years contented with a very moderate salary, supporting himself by private lectures. He made presents to all the academies and public schools of Saxony, and endeavoured by this means every where to excite a predilection for natural philosophy.

Those who were most intimately connected with him, enjoyed his tenderest interest and care.—"In his house," said Boettiger, in his farewell address on the eminence of Gorbitz, "company daily assembled for his advice; and the same hand with which he felt the pulse of nature, raised and supported every unfortunate. His simple manners, his cordial cheerfulness, and his social playfulness, made him the favourite of his fellow-citizens. When Werner entered, every countenance brightened; the women, too, loved the company of a man who, without insipid compliments, always had something delicate and entertaining to say to them. In his earlier years his feeling heart would doubtless have made him highly susceptible of enjoying the sweets of domestic life; but he did not find what he sought. In later years he renounced the idea of them, out of love to science, and was fully indemnified by the cordial attachment of his pupils and friends. Penetrated with that true devotion which worships God in spirit and in truth, he often preached to his pupils the purest morality, which he confirmed by his own example; and even in his lectures often rose with genuine enthusiasm from the miracles of nature to their Divine Author.

Such was the man of whom his country will be always proud; a man equally distinguished by his rare learning, and by his goodness of heart and unspotted character. How just is the grief caused by such a loss! His fairest monument is the gratitude of his pupils, who are spread over all the countries of the world. But his doctrines and his life will not fail of public acknowledgment and praise. This tribute will be given him from France, England, and Italy. Neither must the tongue of his pupils in Germany be mute. Let Von Leonhard dedicate to him his second Lecture in the Academy at Munich! Let Steffens, Ullmann, Hausmann, Mohs, Moll, Linke, and Weiss, and, above all, the feeling Schubert, speak of him! May Gilbert, who defended him against the violent Chenevix, erect a memorial to him in his Annals!—Nor can we doubt but some monument of marble or bronze will be raised to his memory, to which British gratitude and generosity will gladly subscribe, and Frieberg allot a suitable situation to be inclosed for the purpose. For the present, we hope that Böhme, or Buchhorn, will engrave the fine portrait of him, by G. Von Kugelchen, in Dresden, which was intended for his museum, for the satisfaction of his numerous scholars and friends. His most glorious monument, however, will always be the Mineralogical Academy, preserved in uninterrupted activity by his worthy scholars; that academy which he himself sometimes called his beloved daughter, and richly endowed; those who go thither on a pilgrimage, those who there receive instruction, will pay continued homage to the manes of Werner!—*Literary Gazette.*

CRITICAL

